# (3*S*,4*S*)-1-Benzyl­pyrrolidine-3,4-diol

**DOI:** 10.1107/S1600536809055391

**Published:** 2010-03-27

**Authors:** Li-Hua Lu, Xiao-Li Sun, Ping-An Wang

**Affiliations:** a Department of Chemistry, School of Pharmacy, Fourth Military Medical University, Changle West Road 17, 710032, Xi-An, People’s Republic of China

## Abstract

In the title compound, C_11_H_15_NO_2_, the pyrrolidine ring adapts a twisted envelope conformation and the two hydroxyl groups are arranged in a *trans* conformation. The crystal packing is stabilized by inter­molecular O—H⋯N and O—H⋯O hydrogen bonds. A weak C—H⋯π inter­action also occurs.

## Related literature

For the preparation of the title compound, see: Nagel *et al.* (1984 ▶); Inoguchi *et al.* (1990 ▶). The title compound is used in the preparation of the chiral phosphine ligand DEGphos, (+)-(3*R*,4*R*)-*N*-benzyl-3,4-bis­(diphenyl­phosphino)pyrrolidine, (Nagel *et al.*, 1984 ▶), an efficient ligand for Rh-catalysed asymmetric hydrogenation (Tang & Zhang, 2003 ▶).
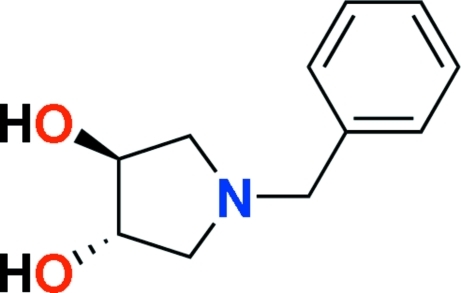


## Experimental

### 

#### Crystal data

C_11_H_15_NO_2_
*M* *_r_* = 193.24
Monoclinic, 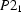

*a* = 6.0244 (10) Å
*b* = 8.1033 (14) Å
*c* = 10.3981 (18) Å
β = 96.016 (2)°
*V* = 504.81 (15) Å^3^
*Z* = 2
Mo *K*α radiation
μ = 0.09 mm^−1^
*T* = 293 K
0.31 × 0.27 × 0.14 mm

#### Data collection

Bruker APEXII CCD diffractometer
Absorption correction: multi-scan (*SADABS*; Bruker, 2005 ▶) *T*_min_ = 0.973, *T*_max_ = 0.987
1440 measured reflections
1440 independent reflections
1348 reflections with *I* > 2σ(*I*)

#### Refinement

*R*[*F* ^2^ > 2σ(*F* ^2^)] = 0.032
*wR*(*F* ^2^) = 0.105
*S* = 1.03
1440 reflections
125 parameters
1 restraint
H-atom parameters constrained
Δρ_max_ = 0.15 e Å^−3^
Δρ_min_ = −0.11 e Å^−3^

### 

Data collection: *APEX2* (Bruker, 2008 ▶); cell refinement: *SAINT* (Bruker); data reduction: *SAINT*; program(s) used to solve structure: *SHELXS97* (Sheldrick, 2008 ▶); program(s) used to refine structure: *SHELXL97* (Sheldrick, 2008 ▶); molecular graphics: *SHELXTL* (Sheldrick, 2008 ▶); software used to prepare material for publication: *Mercury* (Macrae *et al.*, 2006 ▶) and *CAMERON* (Watkin *et al.*, 1996 ▶).

## Supplementary Material

Crystal structure: contains datablocks I, global. DOI: 10.1107/S1600536809055391/jj2016sup1.cif

Structure factors: contains datablocks I. DOI: 10.1107/S1600536809055391/jj2016Isup2.hkl

Additional supplementary materials:  crystallographic information; 3D view; checkCIF report

## Figures and Tables

**Table 1:** Hydrogen-bond geometry (Å, °) *Cg*2 is the centroid of the C1–C6 ring.

*D*—H⋯*A*	*D*—H	H⋯*A*	*D*⋯*A*	*D*—H⋯*A*
O1—H1⋯N1^i^	0.82	2.13	2.918 (2)	162
O2—H2⋯O1^ii^	0.82	2.14	2.914 (2)	157
C10—H10⋯*Cg*2^iii^	0.98	2.86	3.771 (2)	155

Symmetry codes: (i) 
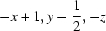
; (ii) 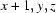; (iii) 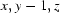.

**Fig. 1.**
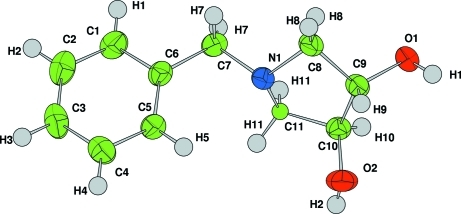
The molecular structure of (I) showing displacement ellipsoids drawn at the 50% probability level. The hydrogen atoms are drawn as spheres of arbitrary radius.

**Fig. 2.**
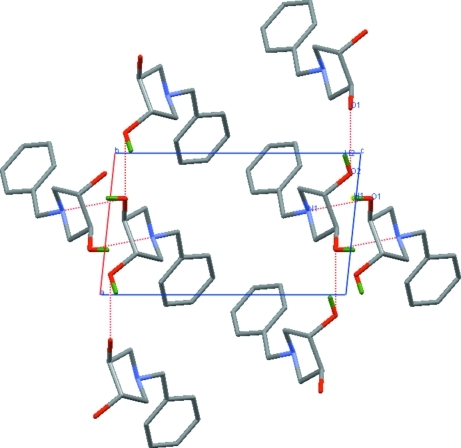
The packing of (I) viewed down the b axis. Dashed lines indicate hydrogen bonds.

## supplementary crystallographic information

### Comment

The title compound (+)-(3*S*,4*S*)-1-benzylpyrrolidine-3,4-diol was obtained from *L*-tartaric acid by condensation with benzylamine followed by reduction with NaBH_4_—BF_3_.Et_2_O. This is used for preparation of the chiral phosphine ligand DEGphos ((+)-(3*R*,4*R*)-*N*-benzyl-3,4- bis(diphenylphosphino)pyrrolidine, (Nagel *et al.*, 1984), an efficient ligand for Rh-catalyzed asymmetric hydrogenations (Tang & Zhang, 2003).

In the title compound, C_11_H_15_NO_2_, the pyrrolidine ring adapts a twisted envelope formation.  The two hydroxyl groups at C9 and C10 are arranged in a *trans-* conformation.  The dihedral angle between the mean planes of the pyrrolidine phenyl rings is 62.4 (2)°.  Crystal packing is stabilized by intermolecular O—H···N and O—H···O hydrogen bonds interactions.  A weak C—H···Cg2 π ring intermolecular intereaction is also observed, where Cg2 = C1–C6.

### Experimental

The synthesis of the title compound is described by Nagel *et al.* (1984). Crystals were grown from its solution in acetone; m.p. 371–373 K.

### Refinement

The absolute structure could not be established from the dffraction data and was assigned based on L-tartaric acid the starting material.

All the H atoms were located in difference Fourier maps. However, they were constrained by riding model approximation. *C*—H_methyl_=0.97 Å; *C*—H_aryl_=0.93 Å; *U*_iso_H_methyl_ and *U*_iso_H_aryl_ are both 1.2 U _eq_(C). O—H is 0.82Å with *U*_iso_(H)=1.5*U*_eq_(O).

### Crystal data

**Table:**

C_11_H_15_NO_2_	*F*(000) = 208
*M**_r_* = 193.24	*D*_x_ = 1.271 Mg m^−^^3^
Monoclinic, *P*2_1_	Mo *K*α radiation, λ = 0.71073 Å
Hall symbol: P 2yb	Cell parameters from 1279 reflections
*a* = 6.0244 (10) Å	θ = 3.2–25.6°
*b* = 8.1033 (14) Å	µ = 0.09 mm^−^^1^
*c* = 10.3981 (18) Å	*T* = 293 K
β = 96.016 (2)°	Block, colorless
*V* = 504.81 (15) Å^3^	0.31 × 0.27 × 0.14 mm
*Z* = 2	

### Data collection

**Table:**

Bruker APEXII CCD diffractometer	1440 independent reflections
Radiation source: fine-focus sealed tube	1348 reflections with *I* > 2σ(*I*)
graphite	*R*_int_ = 0.0000
φ and ω scans	θ_max_ = 25.1°, θ_min_ = 2.0°
Absorption correction: multi-scan (*SADABS*; Bruker, 2005)	*h* = −7→7
*T*_min_ = 0.973, *T*_max_ = 0.987	*k* = −8→9
1440 measured reflections	*l* = 0→12

### Refinement

**Table:**

Refinement on *F*^2^	Primary atom site location: structure-invariant direct methods
Least-squares matrix: full	Secondary atom site location: difference Fourier map
*R*[*F*^2^ > 2σ(*F*^2^)] = 0.032	Hydrogen site location: inferred from neighbouring sites
*wR*(*F*^2^) = 0.105	H-atom parameters constrained
*S* = 1.03	*w* = 1/[σ^2^(*F*_o_^2^) + (0.080*P*)^2^] where *P* = (*F*_o_^2^ + 2*F*_c_^2^)/3
1440 reflections	(Δ/σ)_max_ < 0.001
125 parameters	Δρ_max_ = 0.15 e Å^−^^3^
1 restraint	Δρ_min_ = −0.11 e Å^−^^3^

### Special details

**Table:**

Geometry. All esds (except the esd in the dihedral angle between two l.s. planes) are estimated using the full covariance matrix. The cell esds are taken into account individually in the estimation of esds in distances, angles and torsion angles; correlations between esds in cell parameters are only used when they are defined by crystal symmetry. An approximate (isotropic) treatment of cell esds is used for estimating esds involving l.s. planes.
Refinement. Refinement of *F*^2^ against ALL reflections. The weighted *R*-factor *wR* and goodness of fit *S* are based on *F*^2^, conventional *R*-factors *R* are based on *F*, with *F* set to zero for negative *F*^2^. The threshold expression of *F*^2^ > σ(*F*^2^) is used only for calculating *R*-factors(gt) *etc*. and is not relevant to the choice of reflections for refinement. *R*-factors based on *F*^2^ are statistically about twice as large as those based on *F*, and *R*- factors based on ALL data will be even larger.

### Fractional atomic coordinates and isotropic or equivalent isotropic displacement parameters (Å^2^)

**Table:**

	*x*	*y*	*z*	*U*_iso_*/*U*_eq_	
N1	0.5935 (2)	−0.00443 (18)	0.18867 (14)	0.0358 (4)	
O1	0.3213 (2)	−0.36878 (18)	0.06145 (13)	0.0451 (4)	
H1	0.3166	−0.4139	−0.0094	0.068*	
O2	0.8566 (2)	−0.2626 (2)	0.03312 (13)	0.0556 (5)	
H2	0.9802	−0.2956	0.0628	0.083*	
C1	0.7231 (4)	0.3493 (3)	0.42248 (18)	0.0511 (6)	
H1A	0.6080	0.3348	0.4744	0.061*	
C2	0.8766 (4)	0.4734 (3)	0.4518 (2)	0.0602 (6)	
H2A	0.8637	0.5423	0.5221	0.072*	
C3	1.0500 (4)	0.4951 (3)	0.3760 (2)	0.0582 (6)	
H3	1.1551	0.5777	0.3958	0.070*	
C4	1.0660 (4)	0.3935 (3)	0.2711 (2)	0.0485 (5)	
H4	1.1814	0.4084	0.2195	0.058*	
C5	0.9109 (3)	0.2693 (3)	0.24219 (17)	0.0420 (5)	
H5	0.9242	0.2010	0.1715	0.050*	
C6	0.7361 (3)	0.2451 (3)	0.31701 (16)	0.0387 (4)	
C7	0.5566 (3)	0.1179 (3)	0.28805 (19)	0.0469 (5)	
H7A	0.5373	0.0596	0.3676	0.056*	
H7B	0.4178	0.1750	0.2615	0.056*	
C8	0.3888 (3)	−0.0952 (3)	0.14674 (19)	0.0395 (5)	
H8A	0.2820	−0.0258	0.0956	0.047*	
H8B	0.3203	−0.1384	0.2201	0.047*	
C9	0.4714 (3)	−0.2340 (2)	0.06555 (16)	0.0359 (4)	
H9	0.4813	−0.1938	−0.0226	0.043*	
C10	0.7077 (3)	−0.2710 (2)	0.13021 (16)	0.0371 (4)	
H10	0.7130	−0.3806	0.1702	0.045*	
C11	0.7500 (3)	−0.1372 (3)	0.23333 (17)	0.0408 (5)	
H11A	0.7195	−0.1779	0.3174	0.049*	
H11B	0.9032	−0.0987	0.2389	0.049*	

### Atomic displacement parameters (Å^2^)

**Table:**

	*U*^11^	*U*^22^	*U*^33^	*U*^12^	*U*^13^	*U*^23^
N1	0.0299 (8)	0.0321 (8)	0.0458 (8)	−0.0001 (7)	0.0055 (6)	−0.0030 (6)
O1	0.0388 (8)	0.0407 (8)	0.0567 (7)	−0.0100 (6)	0.0099 (6)	−0.0062 (6)
O2	0.0317 (7)	0.0791 (12)	0.0574 (8)	0.0028 (8)	0.0111 (6)	−0.0130 (8)
C1	0.0573 (13)	0.0502 (14)	0.0466 (11)	0.0031 (11)	0.0097 (9)	−0.0049 (9)
C2	0.0732 (16)	0.0519 (15)	0.0531 (12)	0.0015 (12)	−0.0049 (11)	−0.0135 (11)
C3	0.0554 (14)	0.0422 (13)	0.0724 (13)	−0.0059 (11)	−0.0154 (11)	−0.0048 (11)
C4	0.0393 (11)	0.0412 (12)	0.0639 (11)	−0.0001 (9)	0.0010 (9)	0.0071 (10)
C5	0.0413 (11)	0.0373 (11)	0.0479 (9)	0.0021 (9)	0.0068 (8)	−0.0020 (8)
C6	0.0412 (10)	0.0329 (10)	0.0419 (8)	0.0043 (9)	0.0038 (7)	−0.0011 (8)
C7	0.0447 (12)	0.0407 (11)	0.0578 (11)	0.0001 (10)	0.0176 (10)	−0.0067 (10)
C8	0.0293 (10)	0.0364 (11)	0.0527 (10)	0.0002 (8)	0.0043 (8)	−0.0003 (8)
C9	0.0307 (9)	0.0342 (11)	0.0430 (8)	−0.0024 (8)	0.0045 (7)	0.0023 (8)
C10	0.0316 (10)	0.0351 (10)	0.0447 (9)	0.0036 (8)	0.0046 (7)	0.0017 (8)
C11	0.0374 (11)	0.0396 (11)	0.0446 (9)	0.0040 (9)	0.0001 (7)	0.0001 (8)

### Geometric parameters (Å, °)

**Table:**

N1—C8	1.463 (2)	C4—H4	0.9300
N1—C7	1.466 (2)	C5—C6	1.387 (3)
N1—C11	1.473 (3)	C5—H5	0.9300
O1—C9	1.416 (2)	C6—C7	1.501 (3)
O1—H1	0.8200	C7—H7A	0.9700
O2—C10	1.421 (2)	C7—H7B	0.9700
O2—H2	0.8200	C8—C9	1.521 (3)
C1—C2	1.379 (3)	C8—H8A	0.9700
C1—C6	1.393 (3)	C8—H8B	0.9700
C1—H1A	0.9300	C9—C10	1.539 (2)
C2—C3	1.384 (3)	C9—H9	0.9800
C2—H2A	0.9300	C10—C11	1.528 (3)
C3—C4	1.378 (3)	C10—H10	0.9800
C3—H3	0.9300	C11—H11A	0.9700
C4—C5	1.385 (3)	C11—H11B	0.9700
			
C8—N1—C7	111.39 (14)	C6—C7—H7B	108.2
C8—N1—C11	102.61 (14)	H7A—C7—H7B	107.3
C7—N1—C11	114.28 (14)	N1—C8—C9	102.85 (15)
C9—O1—H1	109.5	N1—C8—H8A	111.2
C10—O2—H2	109.5	C9—C8—H8A	111.2
C2—C1—C6	121.6 (2)	N1—C8—H8B	111.2
C2—C1—H1A	119.2	C9—C8—H8B	111.2
C6—C1—H1A	119.2	H8A—C8—H8B	109.1
C1—C2—C3	119.7 (2)	O1—C9—C8	109.99 (14)
C1—C2—H2A	120.1	O1—C9—C10	114.91 (16)
C3—C2—H2A	120.1	C8—C9—C10	104.09 (15)
C4—C3—C2	119.6 (2)	O1—C9—H9	109.2
C4—C3—H3	120.2	C8—C9—H9	109.2
C2—C3—H3	120.2	C10—C9—H9	109.2
C3—C4—C5	120.3 (2)	O2—C10—C11	113.17 (16)
C3—C4—H4	119.9	O2—C10—C9	107.72 (13)
C5—C4—H4	119.9	C11—C10—C9	104.29 (15)
C4—C5—C6	121.04 (19)	O2—C10—H10	110.5
C4—C5—H5	119.5	C11—C10—H10	110.5
C6—C5—H5	119.5	C9—C10—H10	110.5
C5—C6—C1	117.69 (19)	N1—C11—C10	104.06 (13)
C5—C6—C7	123.90 (17)	N1—C11—H11A	110.9
C1—C6—C7	118.38 (16)	C10—C11—H11A	110.9
N1—C7—C6	116.51 (14)	N1—C11—H11B	110.9
N1—C7—H7A	108.2	C10—C11—H11B	110.9
C6—C7—H7A	108.2	H11A—C11—H11B	109.0
N1—C7—H7B	108.2		
			
C6—C1—C2—C3	−0.7 (3)	C7—N1—C8—C9	−169.66 (15)
C1—C2—C3—C4	0.7 (4)	C11—N1—C8—C9	−46.96 (17)
C2—C3—C4—C5	−0.6 (3)	N1—C8—C9—O1	155.98 (15)
C3—C4—C5—C6	0.5 (3)	N1—C8—C9—C10	32.37 (18)
C4—C5—C6—C1	−0.4 (3)	O1—C9—C10—O2	112.90 (17)
C4—C5—C6—C7	177.2 (2)	C8—C9—C10—O2	−126.76 (16)
C2—C1—C6—C5	0.5 (3)	O1—C9—C10—C11	−126.60 (16)
C2—C1—C6—C7	−177.25 (19)	C8—C9—C10—C11	−6.25 (19)
C8—N1—C7—C6	−166.25 (16)	C8—N1—C11—C10	42.96 (18)
C11—N1—C7—C6	78.0 (2)	C7—N1—C11—C10	163.69 (15)
C5—C6—C7—N1	11.1 (3)	O2—C10—C11—N1	94.90 (18)
C1—C6—C7—N1	−171.29 (17)	C9—C10—C11—N1	−21.88 (18)

### Hydrogen-bond geometry (Å, °)

**Table:**

Cg2 is the centroid of the C1–C6 ring.

**Table:**

*D*—H···*A*	*D*—H	H···*A*	*D*···*A*	*D*—H···*A*
O1—H1···N1^i^	0.82	2.13	2.918 (2)	162
O2—H2···O1^ii^	0.82	2.14	2.914 (2)	157
C10—H10···Cg2^iii^	0.98	2.86	3.771 (2)	155

Symmetry codes: (i) −*x*+1, *y*−1/2, −*z*; (ii) *x*+1, *y*, *z*; (iii) *x*, *y*−1, *z*.
